# Association between milk consumption in middle age and frailty in later life: The Aichi Workers' cohort study

**DOI:** 10.1111/ggi.14916

**Published:** 2024-06-03

**Authors:** Young Jae Hong, Rei Otsuka, Zean Song, Chisato Fukuda, Rina Tajima, Jingyi Lin, Mizuho Hibino, Mei Kobayashi, Yupeng He, Masaaki Matsunaga, Atsuhiko Ota, Yoshihisa Nakano, Yuanying Li, Koji Tamakoshi, Hiroshi Yatsuya

**Affiliations:** ^1^ Department of Public Health and Health Systems Nagoya University Graduate School of Medicine Nagoya Japan; ^2^ Department of Epidemiology of Aging National Centre for Geriatrics and Gerontology Obu Japan; ^3^ Department of Public Health Fujita Health University School of Medicine Toyoake Japan; ^4^ Department of Nursing Nagoya University School of Health Sciences Nagoya Japan

**Keywords:** cohort study, frailty, healthy lifestyle, middle‐aged, milk

## Abstract

**Aim:**

Several studies have shown that dairy consumption in old age is effective in preventing frailty. However, there is a lack of evidence regarding the association between milk consumption during middle age and the development of frailty in old age. Therefore, we carried out an investigation to explore the association between milk consumption during middle age and development of frailty examined after over 15 years of follow up in a long‐term cohort study in Japan.

**Methods:**

We studied 265 participants aged 60–79 years (212 men and 53 women) in 2018, who participated in both the baseline survey in 2002 and the frailty assessment in 2018. The amount of milk consumption (g/day) at baseline was age‐ and energy‐adjusted, and classified into three categories (no, low and high consumption: 0 g/day, ≤135.86 g/day, >135.86 g/day in men and 0 g/day, ≤126.44 g/day, >126.44 g/day in women). Odds ratios (OR) and 95% confidence intervals (CI) for prefrailty/frailty after adjusting for lifestyles at baseline, stratified by sex, were estimated using logistic regression analysis.

**Results:**

The prevalence of prefrailty/frailty in 2018 was 37.7% and 28.3% in men and women, respectively. Milk consumption categories were inversely associated with the prevalence of prefrailty/frailty in men (OR 0.34, 95% CI 0.14–0.84 in low consumption; OR 0.31, 95% CI 0.10–0.95 in high consumption; *P* < 0.05), but not in women (OR 0.53, 95% CI 0.11–2.65; *P* = 0.44).

**Conclusions:**

In this study, milk intake in middle‐aged men was inversely associated with the prevalence of prefrailty/frailty later in life. **Geriatr Gerontol Int 2024; 24: 700–705**.

## Graphical abstract

Milk consumption in middle age was inversely associated with the prevalence of prefrailty/frailty in later life in men independent of lifestyles, medical histories, and calcium, protein and vitamin D intakes. Confirmation of this health‐protecting effect of milk is warranted in a larger sample of men and women.

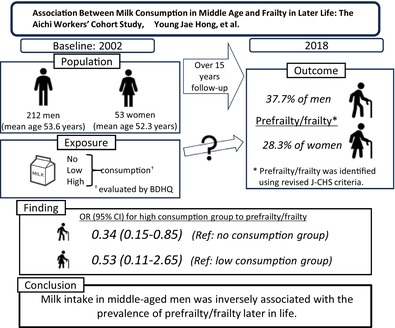

## Introduction

Frailty is a clinical state in which individuals are vulnerable to developing dependency and mortality when exposed to stressors.^1^ It has long been recognized that controlling or preventing the progression of frailty is important to increase healthy life expectancy and maintain a high quality of life.^1^, ^2^, ^3^ Prevention of the development and progression of frailty requires a combination of measures, including diet and exercise,^4^, ^5^, ^6^ and various studies relating such factors in old age with frailty have been carried out.^4^, ^5^, ^7^ However, few studies have investigated the association between lifestyle habits in middle age and the development of frailty in old age. If this association could be identified, it would be possible to develop more fundamental measures to prevent frailty than the current measures of detection and control the progression after old age.

Several studies have shown that high intakes of protein, calcium and vitamin D in old age are effective for preventing frailty.^8^, ^9^, ^10^, ^11^ Some studies have shown that dairy consumption in old age was effective in preventing frailty.^12^, ^13^, ^14^ A previous study suggested that a healthy lifestyle in middle age is a preventive factor for frailty in old age.^15^ Furthermore, high‐milk consumers reportedly engage in healthier lifestyles.^16^ However, to our knowledge, no studies have investigated the association between milk consumption in middle age and frailty in old age.

Therefore, we investigated the association between milk consumption reported in middle age and frailty examined after over 15 years of follow up in a long‐term cohort study in Japan.

## Methods

### Study population

**Figure 1:**
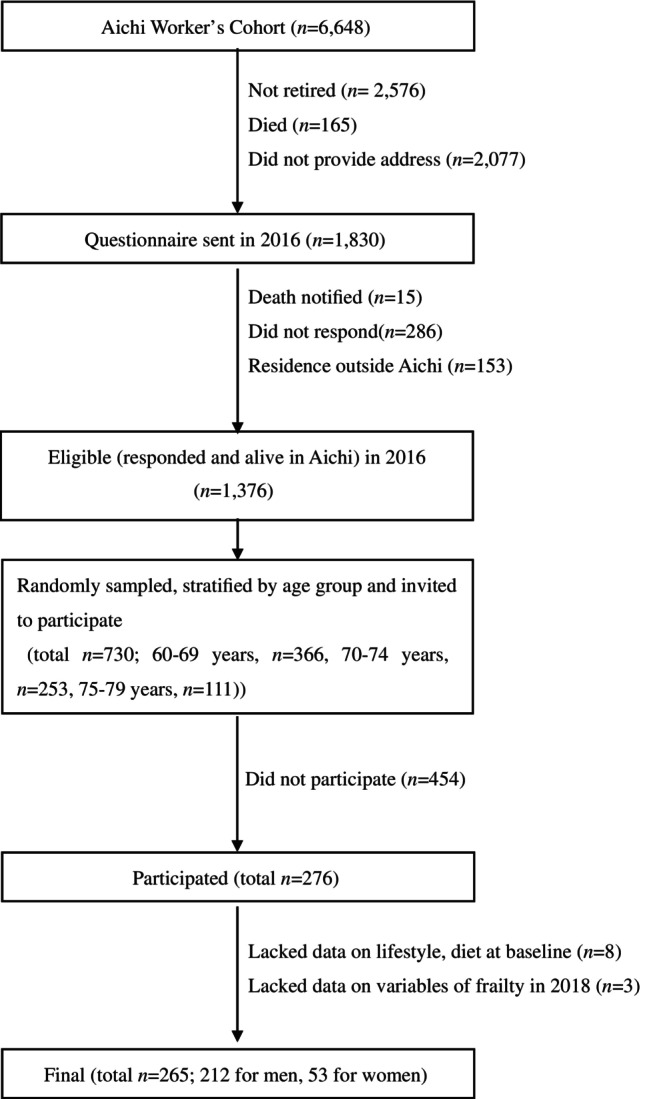
Flowchart for participant selection, Aichi Workers' Cohort Study.

The Aichi Workers' Cohort Study established in 1997 is an ongoing dynamic prospective study on diabetes, cardiovascular diseases and other chronic conditions in the Aichi Prefecture, Japan. The 2002 baseline survey for the cohort was carried out for 6648 civil servants aged 35–66 years. In 2018, we carried out a survey of retirees recruited from the 2002 cohort participants (Fig. 1).

At baseline in 2002, we obtained health checkup data, and self‐reported lifestyle, diet and disease history.

The frailty test was carried out in the 2018 retirees survey. Briefly, 730 persons were randomly selected and invited in three age strata from 1376 eligible retirees: 366 aged 60–69 years, 253 aged 70–74 years and 111 aged 75–79 years. We determined the target sample size of approximately 300. Namely, the target sample size was estimated based on the assumption of detecting the difference in the proportions of frailty/prefrailty among those with (5%) and without (15%) a factor of interest, with alpha and beta errors of 0.05 and 0.20. To reach this target, we randomly selected and invited 730 participants for the survey. Finally, 276 individuals participated in the survey. After excluding eight individuals with missing data on lifestyle and dietary variables in 2002, and three individuals with missing data on frailty in 2018, 265 individuals were included in this analysis (Fig. 1).

We explained the aims and procedures of the study, and obtained written informed consent from all participants. The study protocol was approved by the Bioethics Review Committees of Nagoya University School of Medicine (approval number: 2007–0504) and Fujita Health University (approval number: HM18‐246), Japan.

### Definition of prefrailty/frailty

The revised Japanese version of the Cardiovascular Health Study (the revised J‐CHS) criteria^17^ was used to identify participants with frailty. Briefly, it was defined in 2020 by the Japanese Association on Sarcopenia and Frailty by modifying the original CHS criteria in the USA.^1^ Even though there are various criteria for defining frailty,^18^ we used the revised J‐CHS criteria, as they were developed for use in Japanese older people.^19^ The revised J‐CHS criteria consist of five components (shrinking, exhaustion, weak handgrip, slow walking speed and low physical activity). In the present study, shrinking, exhaustion and physical activity were assessed using questionnaires, whereas the rest were evaluated by tests. First, shrinking and exhaustion were considered as present if participants answered “yes” to the following questions from the Kihon (i.e. basic) Checklist, a kind of questionnaire implemented in Japan to detect individuals with or at risk of frailty in communities by the long‐term care insurance system: “Have you lost 2 kg or more in the past 6 months?” and “In the past 2 weeks, have you felt tired without reason?,” respectively. Physical activity was assessed in the revised J‐CHS using the following two questions: “Do you engage in moderate‐intensity exercise or sports for your health?” and “Do you engage in low‐intensity exercise for your health?” Low physical activity was defined as “no” to both questions. In the present study, we assessed the frequency of light‐, moderate‐ and vigorous‐intensity physical activities during leisure time; that is, light‐intensity activities, such as walking, moderate‐intensity activities, such as brisk walking or gardening, and vigorous‐intensity activities, such as swimming. We calculated the participants’ exercise frequency regardless of intensity, and when the frequency was less than once a week, it was defined as no physical activity.

Low handgrip strength was defined as <28 kg for men and <18 kg for women. Handgrip strength on both sides was measured twice. The mean handgrip strength on the stronger side was used as the participant's handgrip strength. Slow walking speed was defined using a cut‐off (<1.0 m/s) for the usual walking speed. In the survey, participants were requested to walk 5 m at normal speed twice. The time required for each trial was recorded, and the average value was adopted.

The number of the aforementioned five components was counted, and frailty, prefrailty and robust were defined as three to five, one or two and zero components, respectively. In the present study, we combined prefrailty/frailty in the analysis, as only a small number of participants met three to five components.

### Baseline variables

In the 2002 baseline survey, we obtained height and weight data from annual health checkups. Lifestyle information (smoking status and physical activity), dietary information (consumption of milk, alcohol, energy, protein, calcium and vitamin D), histories of lifestyle‐related diseases (hypertension, diabetes, hyperlipidemia and hyperuricemia) and histories of heart diseases, stroke or cancer were obtained using self‐administered questionnaires.

Body mass index (BMI) was calculated as weight (kg) divided by the square of height (m^2^), and was included as a continuous variable in the analysis.

The participants' dietary habits during the preceding month were assessed using a brief‐type self‐administered diet history questionnaire (BDHQ).^20^ The BDHQ was developed to assess the Japanese diet, and has been validated and used for nutritional studies in Japan.^21^ The details of BDHQ's structure, and the method of estimating nutrients have been published.^20^, ^21^ Briefly, the BDHQ consists of four‐page self‐reported questionnaire about the consumption frequency of foods commonly consumed in Japan. Energy and nutrients were calculated using an ad hoc computer algorithm for the BDHQ.^20^

For milk intake, it includes two question items, namely, about low‐fat milk and normal or high‐fat milk. Each question item asks how often participants consume it given the portion size of a cup. The possible responses are as follows: two times or more per day, once a day, four to six times a week, two or three times a week, once a week, less than once a week and no consumption. In the present analysis, we used milk consumption (g/day) estimated by the BDHQ, irrespective of fat content. The estimated amount of milk consumption stratified by sex, except for those in the no consumption group, was adjusted for age and total energy by the residual method. It was classified into three categories (no, low and high consumption: 0 g/day, ≤135.86 g/day, >135.86 g/day in men, and 0 g/day, ≤126.44 g/day, >126.44 g/day in women) and used in the analyses. However, as there were no female participants in the no consumption category, this category was not used in the analysis. In addition, the frequency of milk consumption without being converted to consumption (g) was also analyzed for the association with prefrailty/frailty. In the analysis, the frequency was classified into three categories (<1 cup/week, 1–6 cups/week, ≥7 cups/week).

The BDHQ was used to estimate alcohol, energy, protein, vitamin D and calcium intakes. Alcohol consumption (g/day) was classified into three categories (0, <20, ≥20 g/day). Estimated intakes of protein (g/day), calcium (mg/day) and vitamin D (μg/day), regardless of the sources stratified by sex, were adjusted for age and energy using a residual method, and included as a continuous variable in the analysis.

Smoking status was classified into current, former and never smoking. Physical activity at baseline was assessed based on the number of days engaged in leisure‐time physical activity regardless of intensity. The frequency of physical activity was classified into three categories (<1 day/week, 1–2 day/week, ≥3 day/week) for the analysis. Because the number of female participants was small, the three categories of covariates were collapsed into two. Namely, smoking status was categorized as ever (current or former) and never. Alcohol consumption was categorized as not drinking and drinking. Physical activity was categorized as <1 day/week and ≥1 day/week. Histories of lifestyle‐related diseases were self‐reported for hypertension, hyperlipidemia, diabetes and hyperuricemia. Histories of heart diseases, stroke or cancer were also self‐reported.

### Statistical analysis

Comparisons of age, BMI, energy consumption, protein consumption, calcium consumption and vitamin D consumption among the three consumption categories were carried out using analysis of variance. Comparisons of the proportion of smoking status, alcohol consumption, physical activity, histories of lifestyle‐related diseases, and heart diseases, stroke or cancer among the three groups were carried out using the χ^2^‐test. Odds ratios (ORs) and the 95% confidence intervals (CI) for prefrailty/frailty stratified by sex were estimated using logistic regression analysis. In men, model 1 was adjusted for age, BMI, smoking status, alcohol consumption, physical activity, histories of lifestyle‐related diseases, and heart diseases, stroke or cancer. Model 2 was adjusted for the variables in model 1 plus the consumption of protein, calcium and vitamin D. For women, model 3 was adjusted for age, BMI, smoking status, alcohol consumption, physical activity and lifestyle‐related diseases. As only two female participants had histories of heart diseases, stroke or cancer, this variable was not included. Model 4 was adjusted for the variables in model 3 plus the consumptions of protein, calcium and vitamin D.

As the additional analysis, the frequency of milk consumption and prefrailty/frailty was examined. As there was no robust female participant in the less than one cup of milk consumption categories, the first two milk consumption categories were collapsed for women, leaving two categories (<6 cups/week, and ≥7 cups/week). Statistical analyses were carried out using spss for Windows, Version 29.0 (IBM, Armonk, NY, USA). A two‐sided *P*‐value <0.05 was considered significant.

## Results

The number of participants was 33, 89 and 90 in the no‐, low‐ and high‐milk consumption categories for men, and 26 and 27 in the low and high milk consumption categories for women, respectively. Table 1 presents the baseline characteristics of the participants according to the milk consumption categories. Of 265 participants, 80.0% were men, and the mean age was 53.6 years (SD 3.7) for men and 52.3 years (SD 4.4) for women. There were no significant differences in the following variables among the groups: age, smoking status, alcohol consumption, physical activity, lifestyle‐related diseases, heart diseases, stroke, cancer, energy consumption and vitamin D consumption. Calcium consumption differed significantly among the groups. BMI in women and protein consumption in men differed significantly among the milk consumption categories.

**Table 1:** Baseline characteristics of participants according to milk consumption categories at baseline, Aichi Workers' Cohort Study, 2002, Japan

	Men (*n* = 212)				Women (*n* = 53)			
	No consumption	Low consumption	High consumption	*P*‐value	No consumption	Low consumption	High consumption	*P*‐value
	0 g/day	≤135.86 g/day	>135.86 g/day		0 g/day	≤126.44 g/day	>126.44 g/day	
	*n* = 33	*n* = 89	*n* = 90		*n* = 0	*n* = 26	*n* = 27	
Age, years (SD)	53.9 (3.6)	53.4 (3.6)	53.7 (3.8)	0.69		52.2 (5.2)	52.3 (3.6)	0.93
BMI (SD)	22.8 (2.5)	23.4 (2.8)	23.0 (2.1)	0.36		23.0 (2.6)	21.2 (1.8)	<0.01
Smoking status (%)								
Current	33.3	32.6	16.7	0.11		7.7	7.4	0.68
Former	39.4	34.8	42.2					
Never	27.3	32.6	41.1			92.3	92.6	
Alcohol consumption (%)								
≥20 g/day	48.5	39.3	30.0	0.23		57.7	77.8	0.10
<20 g/day	39.4	51.7	53.3					
0 g/day	12.1	9.0	16.7			42.3	22.2	
Physical activity (%)								
<1 day/week	27.3	40.4	27.8	0.18		42.3	51.9	0.34
1–2 day/week	30.3	31.5	27.8			57.7	48.1	
≥3 day/week	42.4	28.1	44.4					
History of lifestyle‐related diseases (%)	27.3	41.6	40.0	0.33		34.6	18.5	0.16
History of heart diseases, stroke or cancer (%)	3.0	4.5	4.4	0.93		3.8	3.7	0.75
Energy consumption, kcal/day (SD)	1912.8 (585.9)	2068.0 (570.2)	2026.7 (560.4)	0.41		1783.7 (578.8)	1672.6 (521.3)	0.47
Protein consumption^†^, g/day (SD)	68.2 (17.7)	71.9 (14.8)	78.2 (14.2)	0.01		66.3 (13.0)	66.8 (11.7)	0.87
Calcium consumption^†^, mg/day (SD)	437.9 (139.0)	511.7 (147.7)	699.5 (182.6)	<0.01		513.0 (156.7)	670.0 (183.3)	<0.01
Vitamin D consumption^†^, μg/day (SD)	16.5 (11.9)	16.4 (10.5)	17.8 (11.3)	0.68		14.8 (7.8)	14.7 (7.9)	0.98

Abbreviation: SD, standard deviation.

^†^ Adjusted for age and energy using residual method.

The prevalence of prefrailty/frailty was 37.7% in men and 28.3% in women. Table 2 shows the ORs and 95% CIs according to the milk consumption categories stratified by sex. In men, the category with the low and high milk consumption groups was inversely associated with the prevalence of prefrailty/frailty (model 1: OR 0.36, 95% CI 0.15–0.85 in low consumption group; OR 0.35, 95% CI 0.15–0.83 in high consumption group; *P* < 0.05). After adjusting for major milk nutrients, the groups remained inversely associated with the prevalence of prefrailty/frailty (model 2: OR 0.34, 95% CI 0.14–0.84 in low consumption group; OR 0.31, 95% CI 0.10–0.95 in high consumption group; *P* < 0.05). In women, we could not find a significant inverse association between milk consumption and the prevalence of prefrailty/frailty.

**Table 2:** Odds ratios and 95% confidence intervals for prefrailty/frailty according to milk consumption amount categories at baseline, Aichi Workers' Cohort Study, 2002–2018, Japan

	No consumption	Low consumption	High consumption
Men (*n* = 212)	0 g/day	≤135.86 g/day	>135.86 g/day
No. prefrailty or frailty	18/33 (54.5%)	32/57 (36.0%)	30/90 (33.3%)
Model 1	Ref	0.36 (0.15–0.85)	0.35 (0.15–0.83)
Model 2		0.34 (0.14–0.84)	0.31 (0.10–0.95)
Women (*n* = 53)	0 g/day	≤126.44 g/day	>126.44 g/day
No. prefrailty or frailty	0/0 (0%)	9/26 (34.6%)	6/27 (22.2%)
Model 3		Ref	0.37 (0.09–1.54)
Model 4			0.53 (0.11–2.65)

*Note*: Model 1: adjusted for age, body mass index, smoking status, alcohol consumption, physical activity, histories of lifestyle‐related diseases and heart diseases, stroke or cancer. Model 2: adjusted for variables in model 1 plus consumption of protein, calcium and vitamin D. Model 3: adjusted for age, body mass index, smoking status, alcohol consumption, physical activity and history of lifestyle‐related diseases. Model 4: adjusted for variables in model 3 plus consumption of protein, calcium and vitamin D.

Abbreviation: Ref, reference.

In the analyses using milk consumption frequency instead of amount, the most frequent milk consumption category was inversely associated with the prevalence of prefrailty/frailty only in women, which was attenuated after adjusting for nutrients (Table 3).

**Table 3:** Odds ratios and 95% confidence intervals for prefrailty/frailty according to milk consumption frequency categories at baseline, Aichi Workers' Cohort Study, 2002–2018, Japan

	<1 cup/week	1–6 cups/week	≥7 cups/week
Men (*n* = 212)			
No. prefrailty or frailty	24/50 (48.0%)	29/80 (36.3%)	27/82 (32.9%)
Model 1	Ref	0.51 (0.24–1.09)	0.51 (0.24–1.08)
Model 2		0.50 (0.23–1.12)	0.50 (0.17–1.42)
Women (*n* = 53)			
No. prefrailty or frailty	10/25 (40.0%)		5/28 (17.9%)
Model 3	Ref		0.19 (0.41–0.86)
Model 4			0.19 (0.03–1.13)

*Note*: Model 1: adjusted for age, body mass index, smoking status, alcohol consumption, physical activity, histories of lifestyle‐related diseases and heart diseases, stroke or cancer. Model 2: adjusted for variables in model 1 plus consumption of protein, calcium and vitamin D. Model 3: adjusted for age, body mass index, smoking status, alcohol consumption, physical activity and history of lifestyle‐related diseases. Model 4: adjusted for variables in model 3 plus consumption of protein, calcium and vitamin D.

Abbreviation: Ref, reference.

## Discussion

We investigated the association between milk consumption reported in middle age and prefrailty/frailty examined after over 15 years of follow up in a long‐term cohort study in Japan. We found that milk consumption in middle age was inversely associated with prefrailty/frailty in old age. In our analysis adjusted for lifestyle, as well as major nutrients in milk, milk consumption was still inversely associated with prefrailty/frailty in old age. Although there have been several studies on the association between dairy intake and frailty in old age, no studies have examined the association between milk consumption in middle age and frailty in old age.^12^, ^22^

Higher milk consumers reportedly have healthier lifestyles, which could confound the association between milk consumption and frailty.^16^ Previous studies have suggested that a healthy lifestyle, such as non‐smoking and regular exercise in middle age, is a preventive factor for frailty in old age.^15^ Indeed, the present study confirmed that the number of current smokers was lower in the high milk consumption group in men. However, the association between milk consumption and prefrailty/frailty remained significant even after adjusting for lifestyles, including smoking status, alcohol consumption and physical activity. Although the possibility of residual confounding of the other aspects of healthy lifestyles, such as intensity, type, amount and duration of physical activity, cannot be ruled out, our results would suggest that milk consumption itself plays a role in the prevention of frailty.

This, in turn, indicates that nutritional components in milk might explain the inverse association between milk consumption and frailty, although we did not find previous studies investigating the association between nutrient intake in middle age and frailty in old age. In addition, the results are inconsistent in intervention studies in older adults that examined the association between protein intake and frailty.^9^ In the present study, we found that milk consumption remained inversely associated with the prevalence of prefrailty/frailty even after adjusting for the nutrients that make up milk, such as protein, calcium and vitamin D. This result suggested that the major nutritional components of milk might not play important roles in preventing frailty. Milk intake itself or other factors, such as other nutrients or lifestyles associated with milk intake, might have a preventive role for frailty.

The present study had several limitations. First, approximately 40% of those invited actually participated in the 2018 retirees survey. The participants might have been sufficiently healthy to participate in the survey. Alternatively, retirees with health concerns might have participated more frequently in the survey. Because we do not know the actual situation, participants with frailty could be more or less. The proportion of participants with prefrailty/frailty in our study was 34.9%, similar to the proportion of participants with prefrailty and frailty in a previous study.^23^ Based on this finding, concerns regarding selection bias in which participants with frailty are more or less likely to participate do not appear to be significant. Second, only lifestyles and medical histories as of baseline were considered; changes from baseline to 2018 were not considered. Third, although several potential confounding factors were considered, we cannot rule out the possibility of residual confounding by factors that were not included in the present study, in addition to the major nutrients of milk and lifestyles. Fourth, we could not assess participants' frailty status at baseline because of the study design. Therefore, further investigations are required.

In the present study, milk intake in middle‐aged men was inversely associated with the prevalence of prefrailty/frailty later in life.

## Disclosure statement

The authors declare no conflict of interest.

## Author contributions

YJH analyzed the data and wrote the manuscript. YJH and HY designed analyses, interpreted the results, and reviewed and edited the manuscript. RO, YH, MM, AO, YL, KT and HY contributed to the data acquisition. YJH, RO, ZS, CF, RT, JL, MH, MK, YL and HY contributed to the data interpretation. All authors have read and agreed to the published version of the manuscript.

## Data Availability

Data not available due to ethical restrictions.
